# Fall Prevention Using the Fall TIPS Toolkit and Video‐Based Monitoring: A Systematic Review of Clinical Effectiveness and Economic Outcomes

**DOI:** 10.1155/jonm/9948784

**Published:** 2026-11-10

**Authors:** Fatma Al Jabri, Hannele Turunen, Javkhlanbayar Dorjdagva, Minna Mykkänen, Taina Kalliomäki, Antti Hedman, Tarja Tervo-Heikkinen

**Affiliations:** ^1^ Department of Nursing Science Faculty of Health Sciences University of Eastern Finland P. O. Box 1627 Kuopio 70211 Finland uef.fi; ^2^ Wellbeing Services County of North Savo Kuopio Finland; ^3^ Kuopio University Hospital P.O. Box 1711 Kuopio 70211 Finland uef.fi; ^4^ Institute of Public Health and Clinical Nutrition School of Medicine University of Eastern Finland P.O. Box 1627 Kuopio 70211 Finland uef.fi; ^5^ Department of Public Health and Health Policy Hiroshima University Hiroshima 734-8553 Japan hiroshima-u.ac.jp; ^6^ General Services Department Administration Kuopio University Hospital P. O. Box 1711 Kuopio 70211 Finland psshp.fi; ^7^ Client Relations and Quality Assurance Kuopio 70211 Finland

**Keywords:** clinical effectiveness, economic evaluation, Fall TIPS toolkit, fall prevention, healthcare settings, systematic review, video-based monitoring

## Abstract

**Background:**

Falls remain a major patient‐safety and resource challenge. Fall TIPS and video‐based monitoring represent distinct organisational approaches increasingly used to reduce fall risk and support safe care.

**Aim:**

To synthesise evidence on the clinical effectiveness, economic outcomes, and implementation considerations of Fall TIPS and video‐based monitoring for fall prevention in healthcare settings.

**Methods:**

A systematic search of PubMed, Scopus, CINAHL and Web of Science identified studies published between 2014 and 2026. Primary studies of Fall TIPS or video‐based monitoring reporting clinical, economic/resource‐use or implementation outcomes were included. Findings were synthesised narratively following SWiM guidance. Methodological quality was assessed using design‐appropriate JBI tools and MMAT. Reporting completeness of economic evidence was assessed using CHEERS 2022. The review followed PRISMA guidance and was registered in PROSPERO (CRD42024593967).

**Results:**

Twenty‐five studies were included, predominantly from the United States. Fall TIPS studies generally reported reductions in falls and injurious falls, including reductions of 15% and 34%, respectively, in a large acute‐care evaluation. Video‐based monitoring studies also reported reductions in several settings; for example, falls decreased from 6.34 to 5.09 and from 3.9 to 2.8 per 1000 patient‐days in two pre–post studies, although the only RCT found no significant between‐group difference. Economic outcomes were mainly partial, including cost avoidance, budget impact, return‐on‐investment estimates and reduced one‐to‐one sitter use; only one study conducted a full cost–benefit evaluation.

**Conclusion:**

Fall TIPS and video‐based monitoring may improve fall‐related outcomes in selected settings. Several studies also reported operational and resource‐use benefits, particularly reduced reliance on one‐to‐one sitters; however, the economic outcomes were predominantly partial and setting‐specific, and firm conclusions regarding cost‐effectiveness cannot be drawn.

**Implications for Nursing Management:**

Nurse leaders can drive system‐level change by standardising patient selection and escalation protocols, ensuring staff competency, monitoring adherence and outcomes, and integrating safety, privacy, and acceptability into implementation decisions.

## 1. Introduction

Patient falls represent a critical challenge in healthcare, significantly impacting patient safety, care quality and healthcare expenditures [1–3]. Globally, falls rank as the second leading cause of unintentional injury‐related deaths, with associated economic costs exceeding $50 billion annually in 2015 and projected to reach $100 billion by 2030 [4–6]. In United States hospitals alone, approximately 700,000–1 million falls are reported each year, with 30%–50% resulting in significant injuries such as fractures or head trauma [7, 8]. These injuries often lead to extended hospital stays, averaging 6–12 additional days, and increase costs by $17,500 to over $50,000 per incident [9, 10]. The financial burden is further amplified as the Centers for Medicare and Medicaid Services classify fall‐related injuries as preventable hospital‐acquired conditions and do not reimburse the associated costs [11].

In hospitals, falls usually occur during ambulation, repositioning or unsupervised activities. Traditional prevention measures, such as non‐slip socks, bed alarms and bedside sitters, have been inconsistent in their success [12–14]. While bedside sitters can provide direct patient monitoring, their high costs and staffing demands make them unsustainable for many healthcare organisations, with annual sitter expenses reaching millions of dollars in some institutions [15, 16]. Technology‐based interventions have consequently received increasing attention as potential components of broader fall‐prevention strategies [17, 18]. These limitations highlight the need for effective and sustainable organisational approaches to fall prevention.

Two such approaches are the Fall Tailoring Interventions for Patient Safety (Fall TIPS) toolkit and video‐based monitoring systems. Fall TIPS is an evidence‐based, multicomponent programme that combines fall‐risk assessment with individualised prevention plans and actively engages patients, families and healthcare professionals. Previous evaluations have reported reductions of 15%–25% in inpatient falls and 34% in injurious falls [19, 20]. In contrast, video‐based monitoring systems enable trained personnel to observe patients remotely, provide verbal redirection and alert bedside staff when risky behaviour is identified [7, 21]. Some systems may additionally incorporate automated detection or video pattern recognition functions. These monitoring models may reduce reliance on one‐to‐one observation and provide operational and resource‐use benefits.

The effectiveness of these approaches is closely linked to implementation. Reported challenges include staff acceptance, infrastructure and training requirements, communication gaps, and the need for clear escalation pathways and workflow protocols [22, 23]. Evidence regarding long‐term sustainability, scalability and transferability across healthcare settings also remains limited [1, 24]. Although Fall TIPS and video‐based monitoring differ in their theoretical foundations, mechanisms of action and resource requirements, both represent organisational approaches to fall prevention that are relevant to nursing managers and healthcare administrators. Accordingly, they are considered within the same review but synthesised separately.

Several reviews have examined aspects of fall prevention, including sitters and their alternatives [4], virtual sitters [16], staff perceptions of telemonitoring [25], and the use and implementation of Fall TIPS [26]. Recent reviews have further examined surveillance technologies, technology‐enabled interventions, nursing‐led fall‐prevention strategies and emerging fall‐detection technologies [3, 18, 27–29]. However, previous reviews have largely focused on individual interventions, technological effectiveness or implementation perspectives. An integrated synthesis examining clinical effectiveness alongside economic and resource‐use outcomes and implementation considerations across both Fall TIPS and video‐based monitoring remains limited.

Therefore, this systematic review aimed to synthesise evidence on the clinical effectiveness, economic outcomes and implementation considerations of Fall TIPS and video‐based monitoring for fall prevention in healthcare settings.

## 2. Methods

### 2.1. Design

This systematic review was conducted in accordance with relevant guideline recommendations. The reporting of study selection followed the Preferred Reporting Items for Systematic Reviews and Meta‐Analyses (PRISMA) checklist (see Supporting File 1), and the review was reported in line with the PRISMA 2020 statement [30]. Findings were synthesised narratively in accordance with the Synthesis Without Meta‐analysis (SWiM) guidance, using a pre‐specified, stratified approach to address heterogeneity across interventions, outcomes and economic evaluation [31]. The protocol was registered in the International Prospective Register of Systematic Reviews (PROSPERO; CRD42024593967).

### 2.2. Search Strategy

A search strategy for studies evaluating fall‐prevention interventions, including the Fall TIPS toolkit and video‐based monitoring systems, was developed in consultation with an experienced health‐sciences information specialist. PubMed (including MEDLINE, via NCBI), CINAHL (via EBSCOhost), Scopus (via Elsevier) and Web of Science were selected to provide complementary coverage of biomedical, nursing and allied‐health, multidisciplinary, patient‐safety and healthcare‐management literature. The initial search covered the period from January 2014 to October 2024 and was subsequently updated to include studies published up to September 2026. The databases were systematically searched using the strategies presented in Supporting File 2. The reference lists of relevant systematic reviews and other studies were also manually searched. The search strategy included terms across three domains: (1) construct of interest: ‘camera monitoring’, ‘fall prevention’, ‘Fall TIPS model’, ‘pattern recognition system’ and ‘video monitoring’; (2) target population and setting: ‘adult and high‐risk patients’, ‘healthcare settings’ and ‘hospital’; and (3) type of evidence: ‘clinical outcome’, ‘cost analysis’, ‘cost–benefit’, ‘cost‐effectiveness’, ‘cost‐efficiency’ and ‘economic evaluation’.

### 2.3. Eligibility Criteria

This systematic review included articles reporting studies that (a) evaluated fall‐prevention interventions, including video‐based monitoring systems and the Fall TIPS toolkit; (b) reported at least one clinical, economic or implementation‐related outcome, including fall rates, injurious falls, patient safety, resource use, costs, feasibility, acceptability, adherence, staff perceptions or workflow integration; (c) focused on interventions implemented in hospitals, rehabilitation centres or long‐term care facilities; (d) were published between January 2014 and September 2026; and (e) were published in English.

Studies were excluded if they (a) focused on fall‐prevention interventions outside the scope of Fall TIPS or video‐based monitoring; (b) were conducted in non‐clinical or community settings; (c) did not report relevant clinical, economic or implementation outcomes; (d) were published in languages other than English; or (e) were published before 2014. The inclusion and exclusion criteria are presented in Table 1.

**TABLE 1:** Inclusion and exclusion criteria based on the population/setting, intervention, comparison and outcome framework.

Category	Include	Exclude
Population/Setting	1. Adult patients, including medical–surgical patients, older adults and patients at an increased risk of falls. 2. Studies conducted in hospitals, rehabilitation centres, long‐term care facilities and other clinical healthcare settings.	1. Studies conducted exclusively in community or home‐based settings. 2. Studies not involving patients receiving care in clinical healthcare settings.
Intervention	1. Fall TIPS toolkit. 2. Video‐based monitoring interventions for fall prevention, including remote/continuous video monitoring, virtual sitters, automated video monitoring and video pattern‐recognition systems.	Fall‐prevention interventions outside the scope of Fall TIPS or video‐based monitoring, such as exercise programmes, medication reviews, vitamin D supplementation or home‐safety interventions.
Comparator	Usual care, pre‐intervention practice, in‐room sitters, alternative monitoring approaches, or no comparator where appropriate to the study design.	Studies in which the intervention of interest could not be distinguished or evaluated.
Outcome	Studies reporting at least one of the following: (1) Clinical outcomes: falls, injurious falls, fall rates or other patient‐safety outcomes. (2) Economic outcomes: costs, cost avoidance, budget impact, return on investment, sitter/resource utilisation or full economic evaluation. (3) Implementation outcomes: feasibility, acceptability, adherence, staff perceptions, workflow integration, patient selection or implementation processes.	Studies not reporting relevant clinical, economic or implementation‐related outcomes.
Study design	Original empirical studies, including randomised and non‐randomised controlled studies, quasi‐experimental and quality‐improvement studies, cohort studies, cross‐sectional studies, qualitative and mixed‐methods studies, implementation studies and economic evaluations.	Case reports, review articles, commentaries, editorials, protocols without outcome data and other secondary research.

### 2.4. Selection Process

The results of the electronic search were imported into the systematic screening and data‐extraction review tool Covidence. After duplicate removal, two PhD‐level expert reviewers working independently reviewed the titles and abstracts to determine whether each study met the eligibility criteria. The two independent reviewers retrieved full‐text copies of potentially relevant studies and further assessed them according to the inclusion and exclusion criteria. At both stages, disagreements were resolved via discussion. At the end of the full‐text review, the articles that met all predefined criteria were read by two researchers to confirm the inclusion of these articles.

### 2.5. Data Extraction

Data were extracted by one author using a modified version of the Joanna Briggs Institute’s (JBI) Data Extraction Form for Economic Evaluations. The extracted data were subsequently cross‐checked by the second author for accuracy and consistency. Disagreements were resolved through discussion and consultation with a third reviewer. Data were extracted on the study’s setting, evaluation design, participants, intervention used, comparator, economic classification and perspectives, prices and currency used for costing, and time period of analysis. Extracted information was organised into the following outcome categories:

∙ Clinical effectiveness, including fall and injury outcomes (e.g., falls per 1000 patient‐days, falls per 1000 admissions/visits, injurious falls, or percentage change in falls) and implementation‐related clinical metrics where reported (e.g., unattended bed exits).
∙ Partial economic evaluation, including studies that report costs or savings without a formal incremental comparison of both costs and outcomes (e.g., cost saving, cost avoidance, budget impact, return on investment [ROI], sitter‐hour substitution or cost‐minimisation analysis).
• Full economic evaluation, including studies that formally compare at least two alternatives and jointly assess costs and consequences (e.g., cost–benefit analysis, cost‐effectiveness analysis and cost‐utility analysis).

### 2.6. Quality Appraisal, Risk of Bias and Reporting Completeness

Two approaches were used to appraise included studies. Methodological quality and risk of bias were assessed using design‐appropriate JBI critical appraisal tools according to study design (RCT, cohort, quasi‐experimental, analytical cross‐sectional and qualitative) [32]. The mixed‐methods study was appraised using the Mixed‐Methods Appraisal Tool (MMAT) [33]. Two PhD‐level reviewers independently completed the appraisals, and disagreements were resolved through discussion and consensus (Supporting File 3). No studies were excluded based on appraisal, and assessments were used to inform interpretation of findings.

Reporting completeness of economic evaluation was assessed using the Consolidated Health Economic Evaluation Reporting Standards (CHEERS) 2022 checklist [34], which includes 28 reporting items across six domains. CHEERS was used to evaluate transparency of reporting (e.g., perspective, time horizon, price year and costing methods) and was not treated as a risk‐of‐bias tool. For the single study that met criteria for a full economic evaluation (cost–benefit analysis), the Evers Consensus on Health Economic Criteria (CHEC)‐List was additionally applied to appraise the economic component [35]; items were recorded as present/absent to indicate completeness.

### 2.7. Data Synthesis

The findings were grouped by intervention mechanism to avoid combining the Fall TIPS toolkit, a behavioural and engagement intervention, with video‐based monitoring, which operates through surveillance and redirection. Outcomes were reported in the original units used by each study, such as falls per 1000 patient‐days, falls per 1000 admissions or percentage change, and the direction of effect was indicated using effect‐direction signals.

The included studies were grouped into the following intervention classes:

∙ Fall TIPS: a multicomponent behavioural and engagement toolkit focused on risk communication, individualised prevention plans and patient and family engagement; and
∙ Video‐based monitoring: models operating primarily through surveillance, redirection, escalation and workflow support. These included centralised monitoring hubs, mobile or bedside carts, and hybrid models implemented across acute care, rehabilitation, rural hospitals, memory‐care facilities and emergency departments.

A harmonised outcome table was developed to present baseline and follow‐up values, absolute and relative changes, and effect direction where data were available, stratified by outcome unit (Supporting File 4).

Differences in study design, care setting, delivery model, follow‐up duration and outcome measurement precluded meaningful meta‐analysis or statistical subgroup analysis. These sources of heterogeneity were therefore explored through a structured narrative comparison following synthesis without meta‐analysis (SWiM) guidance.

### 2.8. Companion and Overlapping Assessment

Potentially overlapping articles were identified and grouped by health system, setting/units, calendar period and intervention mode. Where multiple publications originated from the same implementation programme or overlapping dataset, only the designated primary effectiveness publication contributed clinical‐effectiveness outcomes to the synthesis. Companion publications were retained only for non‐overlapping implementation, acceptability, process or economic information and were not counted as independent effectiveness evidence.

## 3. Results

### 3.1. Study Selection Process

The results of the selection process are shown in Figure 1. The database search, after duplicates were removed, yielded a total of 271 records. Title and abstract screening identified 45 full‐text articles for eligibility assessment. Following full‐text review, 25 articles met the inclusion criteria and were included in the review in accordance with PRISMA guidelines [30].

**FIGURE 1:**
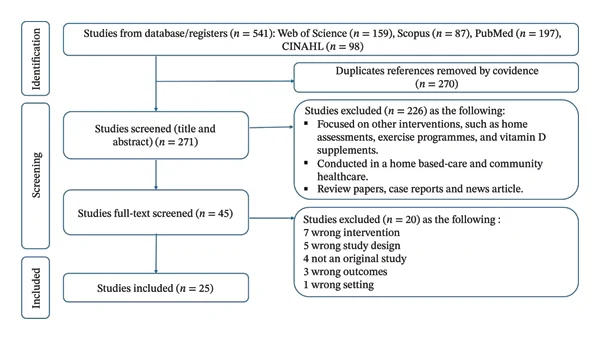
PRISMA flow diagram.

### 3.2. Study Characteristics

Study characteristics are summarised in Table 2. Of the 25 included studies, 23 (92.0%) were conducted in the United States, with one study each from Australia (4.0%) and Brazil (4.0%), indicating limited geographical diversity. The evidence base predominantly evaluated video‐based monitoring, while a smaller number of studies examined Fall TIPS.

**TABLE 2:** Study characteristics.

Author (Year), country	Setting	Study design	Intervention (class + model)	Comparator
*Clinical effectiveness and/or implementation/acceptability evidence*				
Bayen et al. (2017), USA	Memory‐care dementia facility	Pilot observational (before–after)	VM (CVM + video incident review; facility‐based)	Pre‐implementation baseline
Cook et al., (2020), USA	Emergency department	QI (pre–post over time)	Mixed multifactorial programme (includes RVM + alarms + workflow/culture changes; hybrid bundle)	Historic baseline
Daley et al. (2020), USA	Acute neuro‐medical inpatient unit	RCT	VM/ESS (ESS ± dedicated rounder; monitoring model)	ESS with vs without rounder (active comparator)
Dykes et al. (2021), USA	Acute‐care hospitals (med‐surg nurses)	Cross‐sectional survey	Fall TIPS (behaviour/engagement toolkit; perceived efficiency)	Self‐reported comparison to other programmes
Dykes et al. (2020), USA	Acute‐care medical units (multi‐site)	Non‐randomised controlled trial (stepped‐wedge ITS)	Fall TIPS (behaviour/engagement toolkit)	Usual care (pre‐implementation)
Ergai et al. (2023), USA	Multiple hospitals/units	Quasi‐experimental (pre–post) + nurse survey	VM (RVM/VPR telesitter; centralised telesitter model)	Pre‐implementation baseline
Hebb et al. (2021), USA	Acute hospital units	Cross‐sectional survey	VM (CVM vs sitters; acceptability)	In‐person sitters (perception‐based)
Jones et al. (2021), USA	Small rural hospitals	One‐group pre–post	VM (AVMS with alerts; automated monitoring model)	Baseline (no AVMS)
Klymko et al. (2016), USA	Hospital context (staff perspectives)	Qualitative focus groups	VM (VM used as learning/incident understanding tool; model not evaluated)	None
Medeiros and Gonçalves (2024), Brazil	Public teaching hospital	Evaluability study (programme planning)	Fall TIPS (Brazil adaptation; logic model/indicators)	None
Quigley et al. (2019), USA	Multi‐hospital programme (71 hospitals)	Descriptive large‐scale evaluation	VM (PEVS; interactive/patient‐engaged)	Implied traditional sitters/usual care
Sand‐Jecklin et al. (2016), USA	Acute hospital units	Quasi‐experimental (pre–post)	VM (centralised VM room; centralised hub)	Pre‐implementation(sitters + usual care)
Sand‐Jecklin et al. (2019), USA	Acute hospital	Descriptive process evaluation	VM (centralised VM; process/standardisation)	None (no effectiveness comparator)

*Partial economic outcomes (ROI, cost analysis, cost saving, budget impact and cost avoidance)*				
Burtson and Vento (2015), USA	Acute hospital system (multi‐unit)	QI with programme evaluation (pre–post)	VM (mobile VM carts + sitter protocol; mobile model)	Traditional 1:1 sitters (baseline)
Cournan et al. (2018), USA	Inpatient rehabilitation hospital	Sequential cohort (pre–post)	VM (centralised monitoring + cameras; centralised hub)	Usual care (sitters/alarms)
Davis and Carter‐Templeton (2020), USA	Orthopaedic inpatient unit	QI (pre–post)	VM (two‐way video observation; model not fully specified)	Baseline period/usual care bundle
Dykes et al. (2017), USA	Acute‐care units (two medical centres)	QI pilot (pre–post)	Fall TIPS (toolkit; paper/laminated/HIT‐supported)	Pre‐implementation
Kalafus et al. (2025), USA	Long‐term acute‐care hospital (LTACH)	Prospective observational cohort (historical comparator)	VM (CVM; centralised telesitter model)	Historical pre‐implementation period (1:1 sitter/usual care)
Kowalski et al. (2018), USA	Government healthcare facility (inpatient)	QI with budget‐impact projection	VM (VMS replacing sitters; central monitoring concept)	Traditional 1:1 sitter model
Purvis et al. (2018), USA	Academic medical centre	QI with programme implementation	VM (CO programme with VM; centralised workflow)	PSA (1:1 observation model)
Quigley et al. (2022), USA	Hospital setting (adult inpatients)	QI with programme evaluation (pre–post)	VM (CVM virtual sitters; model varies)	Pre‐implementation (sitters only)
Sosa et al. (2024), USA	Tertiary academic hospital	Retrospective cohort (historical control)	VM (two‐way VM + sitters; hybrid model)	Pre‐implementation (sitters only)
Votruba et al. (2016), USA	Urban hospital units	Prospective QI (pre–post)	VM (telesitter up to 12 pts; centralised telesitter)	Baseline 1:1 companions
Woltsche et al. (2022), Australia	Geriatric/aged care wards	Prospective cohort/QI	VM (portable VM ‘baby monitor’; portable model)	Pre‐implementation (usual care)

*Full economic evaluation (cost–benefit)*				
Dykes et al. (2023), USA	Multi‐hospital med‐surg units	ITS + matched case–control (economic analysis)	Fall TIPS (behaviour/engagement toolkit; implementation programme)	Pre‐implementation (no Fall TIPS)

*Note:* Fall TIPS, Fall Tailoring Interventions for Patient Safety. VM, video‐based monitoring; CVM, continuous video‐based monitoring; VMS, video‐based monitoring system; PEVS, patient‐engaged video‐based surveillance; pts, patients.

Abbreviations: AVMS, automated video monitoring system; ESS, electronic surveillance system; HIT, health information technology; ITS, interrupted time series; med‐surg, medical–surgical; PSA, patient safety attendant; QI, quality improvement; RCT, randomised controlled trial; ROI, return on investment; RVM, remote video‐based monitoring; VPR, video pattern recognition.

Thirteen studies (52.0%) reported clinical and/or implementation outcomes without monetised costs and primarily used quality improvement or observational designs; only one randomised controlled trial was identified. Eleven studies (44.0%) contributed partial economic evidence, typically reporting sitter substitution, cost avoidance, budget impact, return on investment or other resource‐use outcomes, mainly from a healthcare provider or hospital perspective. Only one study (4.0%) met the criteria for a full economic evaluation and conducted a cost–benefit analysis [20].

Economic reporting varied considerably across studies. Only two studies reported a price year [20, 21], while discounting was either not reported or not applicable. Capital, operational and implementation costs were also reported inconsistently across studies [7, 21, 36–38], limiting the comparability and standardisation of cost estimates across settings.

### 3.3. Methodological Quality and Risk of Bias

Across the 25 included studies, most employed quasi‐experimental/quality‐improvement pre–post designs (*n* = 14, 56.0%), followed by analytical cross‐sectional surveys (*n* = 4, 16.0%), qualitative studies (*n* = 2, 8.0%) and cohort studies (*n* = 2, 8.0%). One was an RCT (4.0%) and one was a mixed‐methods process evaluation (4.0%; appraised with the MMAT). One study additionally met the criteria for a full economic evaluation. Most pre–post studies demonstrated clear intervention–outcome direction and consistent outcome definitions; however, the absence of concurrent control groups, co‐interventions occurring alongside video‐based monitoring and limited adjustment for confounding were common methodological limitations.

Outcome measurement frequently relied on routine incident‐reporting systems, raising concerns about potential underreporting or measurement reliability. Statistical rigour varied, with some studies applying appropriate inferential analyses (e.g., rate comparisons or regression modelling), while others reported descriptive trends without formal hypothesis testing.

The cross‐sectional survey studies generally provided adequate descriptions of settings and participants and used appropriate statistical approaches, although nonresponse bias and limited multivariable adjustment were recurrent concerns. The mixed‐methods process evaluation demonstrated effective integration of qualitative and quantitative findings to inform implementation improvements, but provided limited reporting on methodological rigour procedures.

### 3.4. Reporting Completeness

The CHEERS 2022 checklist was used to assess the transparency and completeness of economic reporting in studies contributing economic evidence. CHEERS was used as a reporting guideline and not as a tool for assessing methodological quality or risk of bias. Studies reporting only clinical or implementation outcomes without economic evidence were not assessed using CHEERS. Detailed item‐level reporting is provided in Supporting File 5, with each applicable item classified as Reported (R) or Not Reported (NR).

Across studies contributing economic evidence, core information such as study population, setting, time horizon, outcome selection and measurement, and summary of findings was generally well reported. In contrast, several economic‐specific items were reported less consistently, particularly the health economic analysis plan, discounting, currency and price year, measurement and valuation of resources and costs, and the rationale and description of economic models. These reporting gaps limited the comparability and transferability of economic findings across settings.

The methodological quality of the only full economic evaluation included in the review [20] was additionally assessed using the Evers CHEC‐List and is presented in Supporting File 6. The study met 18 of 19 applicable criteria, indicating high methodological quality. It clearly defined the study population, comparator, research question, perspective and time horizon; appropriately identified, measured and valued costs and outcomes; conducted incremental analysis and sensitivity analysis; and addressed generalisability, conflicts of interest, and ethical or distributional considerations. The only CHEC‐List item considered not applicable was discounting of future costs and outcomes, consistent with the study’s analytic time frame.

### 3.5. Companion and Overlapping Publications

Potentially overlapping publications were identified and grouped by health system, setting, study period and intervention. Two publication clusters were identified (Supporting File 7): (a) Sand‐Jecklin et al. [8] was designated as the primary effectiveness publication, with Sand‐Jecklin et al. [22] contributing process and standardisation evidence; and (b) Dykes et al. [1] was designated as the primary effectiveness publication for Fall TIPS, with companion publications contributing implementation and adherence [19], acceptability and time‐related outcomes [11], and economic evidence [20].

### 3.6. Synthesis of Results

To enhance interpretability and avoid combining mechanistically distinct interventions, evidence from the 25 included studies was synthesised using a pre‐specified, stratified approach by intervention class, delivery model and overall direction of effect. As presented in Table 3, findings were synthesised separately for Fall TIPS, a behavioural and patient‐engagement intervention, and video‐based monitoring and surveillance‐based models, alongside related implementation and acceptability evidence.

**TABLE 3:** Findings by intervention class and synthesis strata.

Mechanism of action	Delivery model	Typical comparator	Included studies	Primary outcome unit	Effect direction
*Fall TIPS (behavioural/engagement) toolkit*					
Risk identification and communication, individualised plans, patient/family engagement, workflow standardisation	Paper‐based and/or EHR/HIT‐enabled toolkit	Usual care (pre‐implementation fall‐prevention practice)	Dykes et al. [19]	Injurious falls/1000 pt‐days	↓
			Dykes et al. [1]	Falls and injurious/1000 pt‐days	↓
			Dykes et al. [11]	Perceived efficiency	↓
			Dykes et al. [20]	Falls and injurious	↓
			Medeiros and Gonçalves [43]	Proposed indicators	NA

*Video-based monitoring (VM)–Centralised hub*					
Continuous remote surveillance, real‐time redirection, escalation to bedside staff, workflow enablement	Central VM room with trained VMTs using fixed and/or portable cameras	1:1 in‐room sitters and/or usual care (pre‐implementation)	Bayen et al. [24]	Percentage fall reduction (facility level)	↓
			Cournan et al. [9]	Falls/1000 pt‐days	↓
			Sand‐Jecklin et al. [8]	Falls/1000 pt‐days	↓
			Sand‐Jecklin et al. [22]	Process outcomes (standardisation focus)	NA
			Quigley et al. [44]	Falls/adverse events	↓
			Quigley et al. [2]	Fall + injury rate	↓
			Purvis et al. [15]	Unit falls	↓
			Hebb et al. [13]	Staff acceptability	↔
			Burtson and Vento [7]	Falls vs benchmark	↔
			Kowalski et al. [37]	Fall/injury outcomes (projection‐based)	↓
			Kalafus et al. [36]	Falls/1000 pt‐days; 1:1 sitter hours	↓

*Video-based monitoring (VM)–Mobile/portable*					
Surveillance via mobile/portable devices with redirection/escalation	Mobile carts, portable/bedside devices	1:1 sitters and/or usual care	Votruba et al. [38]	Falls per discharge	↓
			Woltsche et al. [23]	Falls/bed‐days	↓
			Davis and Carter‐Templeton [45]	Falls/1000 pt‐days	↓

*Video-based monitoring (VM)–Hybrid models*					
VM combined with partial in‐person observation; substitution of sitter coverage where feasible	Two‐way VM + in‐person sitters (hybrid staffing)	Traditional sitter‐only model (baseline)	Sosa et al. [21]	Percentage of patients with falls	↓
			Daley et al. [10]	Falls/1000 pt‐days	↔
			Ergai et al. [40]	Injurious falls	↓ (injury); ↔ (non‐injury)

*AVMS/ESS*					
Automated detection/alerts and/or electronic surveillance to prompt staff response; may include predictive elements	AVMS alerts and/or ESS	Baseline monitoring/no alerts, alternative surveillance workflow	Jones et al. [14]	Falls/1000 admissions	↓

*Multifactorial programmes–Implementation and acceptability*					
Multi‐component fall programme (VM + alarms + workflow redesign + training/culture elements)	Bundle implementation (VM as one component)	Historic baseline	Cook et al. [46]	Falls and injurious falls and visits	↓
Qualitative (implementation/acceptability context)	Focus groups exploring staff experiences and antecedents to falls within a VM context	None	Klymko et al. [42]	Themes (no quantified fall outcome)	NA

*Note:* Fall TIPS, Fall Tailoring Interventions for Patient Safety. VM, video‐based monitoring; VMT, video‐based monitoring technician; AVMS, automated video‐based monitoring system; pt‐days, patient‐days; pt, patient; 1:1, one‐to‐one (continuous in‐room observation); ↓, decrease; ↑, increase; ↔, no meaningful change.

Abbreviations: EHR, electronic health record; ESS, electronic surveillance system; HIT, health information technology; NA, not available/applicable.

Overall, Fall TIPS effectiveness studies generally reported reductions in falls and/or injurious falls in acute‐care settings, while companion publications contributed implementation, acceptability and economic evidence. Most video‐based monitoring studies used centralised hub models and generally reported reductions in falls and/or injuries, although some reported no meaningful change in fall rates or focused primarily on acceptability. Mobile or portable monitoring showed consistent directional reductions across three studies. Findings from hybrid models were mixed, including positive findings in one cohort study, no significant between‐group difference in one RCT and reduced injurious falls but unchanged non‐injurious falls in another study. Automated or electronic surveillance and a multifactorial emergency department bundle incorporating video‐based monitoring also reported reductions in falls. Qualitative studies contributed implementation and acceptability insights rather than quantified effectiveness outcomes.

Substantial heterogeneity was observed in outcome denominators, including falls per 1000 patient‐days, per 1000 admissions, per visit and percentage‐based measures (Table 3). To improve interpretability, a harmonised outcomes table (Supporting File 4) presents outcomes in their original units and, where available, baseline and follow‐up values with absolute and relative changes. Across outcome units, findings generally indicated directional reductions; however, variation in denominators, baseline risk and reporting limited direct comparison. Several studies reported relative changes without corresponding baseline or follow‐up values, further limiting interpretation of absolute effect sizes. Findings were therefore synthesised descriptively within comparable outcome units rather than pooled.

Building on the intervention classes and synthesis strata in Tables 3 and 4 provides a summary of cost and effectiveness metrics of included studies. The outcomes are organised by clinical effectiveness, partial economic evaluation, full economic evaluation and implementation/acceptability:

**TABLE 4:** Summary of cost and effectiveness metrics.

Author (year)	Key cost metric	Key effectiveness metric
*Fall TIPS toolkit*		
Dykes et al. (2017)	Programme cost: US$4600 (30‐bed unit)	Injurious falls: 1.00 ⟶ 0.54/1000 patient days, adherence: > 80%
Dykes et al. (2020)	Not measured (injury cost referenced from literature)	Falls: ↓ 15%, injurious falls: ↓ 34%
Dykes et al. (2021)	Nurse time saved: 10.7 min/patient	Falls: ↓ 25%, injurious falls: ↓ 34%, high perceived value
Dykes et al. (2023)	Total cost savings: US$22M	Falls: ↓ 19%, injurious falls: ↓ 20%
Medeiros and Gonçalves (2024)	Not measured	No effectiveness outcomes

*Video-based monitoring (VM)*		
Bayen et al. (2017)	Cost savings inferred (no costing), equipment listed only	Falls: ↓ 79%
Burtson and Vento (2015)	ROI: 29.2, savings: US$1,718,823/year, sitter staffing: ↓ 53.6%	Falls: ↔ (vs benchmark)
Cook et al. (2020)	Not quantified	ED falls/1000 visits: 0.73 ⟶ 0.55, injury: 0.09 ⟶ 0.03
Cournan et al. (2018)	Sitter savings: US$186,120, fall‐expense savings: US$40,000	Falls/1000 patient days: 6.34 ⟶ 5.09
Daley et al. (2020)	Not measured	Falls/1000 patient days: 1.02 (rounder) vs 3.05 (no rounder)
Davis and Carter‐Templeton (2020)	Savings: US$88,000 (falls avoided + sitters reduction)	Falls/1000 patient days: 2.6 ⟶ 0
Ergai et al. (2023)	Resource inputs described (FTE/equipment), no cost results	Injurious falls: ↓ 39.15% (*p* = 0.006)
Hebb et al. (2021)	Not costed	Effectiveness was perceived as low (12.73% rated as effective as sitters)
Jones et al. (2021)	Equipment + technician costs	Sensitivity: 97.4%, lead time: 28 s, ↓ unassisted falls
Kalafus et al. (2025)	Estimated total cost savings: US$3,201,624 including fall costs; US$160,234 excluding fall costs	Falls/1000 patient‐days: 4.9 ⟶ 3.8 (↓ 25.1%, *p* = 0.02); 1:1 sitter hours/month: 1428 ⟶ 140 (↓ 90.2%, *p* < 0.001)
Klymko et al. (2016)	Not measured	No quantified fall outcomes
Kowalski et al. (2018)	Cost avoidance: US$368,428 (year 1)	Fall‐related cost: ↓ 50%, sitter costs: ↓ 75%
Purvis et al. (2018)	PSA FTE cost reduction: US$210,912/year	Falls: 1 vs 8 (pre–post, per programme report), VM usage: 37%
Quigley et al. (2022)	Sitter substitution savings: US$97,000	Falls: ↓ 14%, injuries: ↓ 6%
Quigley et al. (2019)	Sitter substitution + implementation cost (reported)	Adverse events (incl. falls): ↓
Sand‐Jecklin et al. (2016)	Sitter shifts: ↓ 23.2% (VMT shifts added)	Falls/1000 patient days: 3.9 ⟶ 2.8
Sand‐Jecklin et al. (2019)	Savings implied (no analysis)	Falls: ↓ 28.5%, sitter use: ↓ 23.2%
Sosa et al. (2024)	Savings: > US$800,000/year, hardware: US$14,561/15 pts	Falls: 3.5% ⟶ 2.7% (OR 0.49, *p* < 0.001)
Votruba et al. (2016)	Avoided fall costs (US$52k–US$87.5k) + sitter (US$25.2k), telesitter cost: US$120k/year	Falls per discharge: 1.7% ⟶ 1.1% (↓ 35%)
Woltsche et al. (2022)	Equipment only AU$480 (2 cameras + monitor)	Overnight falls: ↓ (directional, rate per bed‐days in article)

*Note:* Fall TIPS, Fall Tailoring Interventions for Patient Safety. VM, video‐based monitoring; VMT, video‐based monitoring technician; pts, patients; *p*, *p*‐value; sec, seconds; min, minutes; ↓, decrease; ↑, increase; ↔, no meaningful change;/1000 patient days, per 1000 patient‐days.

Abbreviations: ED, emergency department; FTE, full‐time equivalent; OR, odds ratio; PSA, patient safety attendant; ROI, return on investment.

#### 3.6.1. Clinical Effectiveness

Across intervention classes, most studies reported directional reductions in falls and/or injurious falls, although outcome units and denominators varied substantially (Table 3, Supporting File 4). For Fall TIPS, the primary effectiveness evaluation reported reductions in falls and injurious falls following implementation [1]. Companion publications contributed additional information on implementation and adherence [19], perceived efficacy and acceptability [11], and economic outcomes [20] but were not treated as independent effectiveness evidence. For video‐based monitoring studies, reductions were reported across delivery models in several settings; however, effects were not uniform, and the only RCT reported no significant between‐group difference [10]. Overall, the findings are presented descriptively rather than pooled due to heterogeneity in the denominators, follow‐up periods and study designs (Supporting File 4).

#### 3.6.2. Cost Analysis and Partial Economic Evaluation

The economic evaluations were predominantly partial (cost savings/avoidance, ROI or budget impact), usually from a provider/hospital perspective, and most frequently linked to reduced reliance on a 1:1 sitter ratio and constant observation (Table 4). Costing methods and transparency varied widely across studies (e.g., incomplete reporting of capital versus operational costs, training/implementation inputs and valuation assumptions), limiting cross‐study comparability (Tables 2 and 4). Several studies reported large operational savings or favourable ROI, driven primarily by staffing substitution; however, these should be interpreted as setting‐specific signals rather than transferable estimates because price year and discounting were rarely reported.

#### 3.6.3. Full Economic Evaluation

Only one study met criteria for a full economic evaluation (cost–benefit analysis), linking observed changes in falls and injurious falls to avoided costs under stated assumptions and exploring uncertainty via sensitivity analysis [20]. Accordingly, conclusions about ‘cost‐effectiveness’ in the formal health‐economic sense are not supported for the broader evidence base.

#### 3.6.4. Implementation, Acceptability and Workflow Integration

Most studies emphasised implementation and operational fit rather than monetised outcomes. Recurring determinants of success included clear eligibility/patient selection, standardised initiation/discontinuation workflows, reliable escalation pathways and training/communication between monitoring staff and bedside teams (e.g., process‐focused evaluations and acceptability studies). Staff acceptance was mixed across settings, and qualitative evidence highlighted that video‐based monitoring can serve as both a prevention strategy and a learning mechanism for understanding fall circumstances and workflow vulnerabilities, reinforcing the importance of implementation design alongside effectiveness measurement.

## 4. Discussion

This systematic review evaluated two mechanistically distinct fall‐prevention approaches and synthesised their evidence separately. Fall TIPS studies generally reported consistent reductions in falls and/or injurious falls in acute‐care settings, whereas findings for video‐based monitoring were more variable across delivery models and care settings. No effects were pooled across the two intervention classes.

### 4.1. Clinical Effectiveness and Injury Prevention

Across settings, multiple studies reported reductions in falls and/or injurious falls following implementation of the Fall TIPS toolkit or video‐based monitoring, including work conducted in high‐risk populations such as older adults and people with cognitive impairment in memory‐care and long‐term acute‐care settings [21, 24, 36]. For the Fall TIPS toolkit, studies emphasised structured risk communication and co‐produced prevention plans, which may strengthen adherence to prevention actions and reinforce shared responsibility among staff, patients and their families [11]. This interpretation is consistent with broader evidence indicating that effective inpatient fall prevention is multifactorial and may depend on combinations of patient education, nursing leadership and organisational approaches to change [39].

For video‐based monitoring, effectiveness signals were reported across different delivery models, including centralised hubs, mobile or portable monitoring, hybrid models, and automated or electronic surveillance. However, effects were not uniform and appeared sensitive to implementation choices, such as patient selection criteria and the timeliness and reliability of bedside response pathways [22, 36]. Recent evidence identified promising reductions associated with video monitoring but emphasised substantial methodological heterogeneity [3]. Similarly, Stephen et al. [29] identified three distinct remote‐monitoring models and found that perceived effectiveness varied according to how and why monitoring was implemented. Evidence from the only RCT indicated that adding staff roles, such as rounders, did not necessarily provide additional benefits [10]. Importantly, because outcomes were reported using different units across studies, findings were interpreted based on within‐study absolute or relative changes and effect direction within comparable denominators, rather than through cross‐unit comparisons.

### 4.2. Economic Outcomes

Economic outcomes were reported mainly from a healthcare‐provider or hospital perspective and consisted predominantly of partial evaluations, including cost avoidance, budget impact, return‐on‐investment estimates and reductions in one‐to‐one sitter use [2, 7, 8, 36–38]. Several video‐based monitoring studies reported operational savings associated with reduced reliance on bedside sitters or patient safety attendants, suggesting potential resource‐use benefits where remote monitoring can substitute for continuous one‐to‐one observation [2, 7, 36, 38]. This interpretation is consistent with recent evidence showing that workforce optimisation and cost reduction are important drivers for the adoption of remote video monitoring, particularly in U.S. hospitals [29]. In contrast, only one study, evaluating the Fall TIPS programme, met the criteria for a full economic evaluation and reported a cost–benefit analysis linking reductions in falls and injurious falls with avoided costs [20].

These findings should not be interpreted as evidence of cost‐effectiveness across either intervention class. Most studies did not conduct formal comparative economic evaluations. Incomplete reporting of perspective, price year, resource use and costing methods further limited cross‐study comparability and transferability of reported savings across healthcare settings [34].

### 4.3. Implementation, Workflow Integration and Staff Experience

Implementation factors emerged as important influences on intervention delivery and outcomes across both intervention classes. Studies reporting stronger outcomes typically described clearer eligibility criteria, standardised workflows for initiating and discontinuing monitoring, and reliable communication between monitoring personnel and bedside teams [7, 22, 36]. Conversely, barriers included resistance to replacing in‐room sitters, infrastructure delays, and training gaps, factors associated with variable staff acceptance and perceived usefulness [13, 40]. These findings align with recent evidence highlighting stakeholder training and engagement as important implementation strategies for hospital fall prevention [41].

Staff perceptions of remote video monitoring were also influenced by workload, knowledge, perceived effectiveness and ethical concerns [29]. Beyond direct effectiveness, some studies positioned video‐based monitoring as a learning tool for understanding fall mechanisms and system vulnerabilities, supporting continuous improvement even when causal attribution is uncertain [42]. Altogether, these findings suggest that the impact of both the Fall TIPS toolkit and video‐based monitoring is shaped not only by intervention design but by implementation quality, particularly escalation reliability, staff engagement and workflow integration.

### 4.4. Strengthening the Evidence Base: Priorities for Research and Practice

Future studies should report fall outcomes using clearly defined denominators and baseline risk and improve economic reporting by specifying currency, price year, analytical perspective, and relevant capital and operational costs. Studies should also examine sustainability by accounting for maintenance, training, change‐management and workflow‐redesign requirements. Longer follow‐up periods and multisite evaluations would improve understanding of the durability and transferability of effects. Recent reviews similarly highlight substantial heterogeneity in study designs, clinical settings, intervention components and outcome definitions, which continues to limit comparison and evidence synthesis [3].

From a practice perspective, organisations considering Fall TIPS or video‐based monitoring should approach implementation as a system‐level change involving clear patient‐selection criteria, escalation pathways, communication protocols, staff training and competency requirements, and ongoing monitoring of both patient‐safety and workforce outcomes. This approach is consistent with recent implementation evidence emphasising stakeholder education, relationship‐building and context‐sensitive implementation of hospital fall‐prevention interventions [41].

### 4.5. Equity, Privacy and Acceptability Considerations

Patients with cognitive impairment and other clinical or behavioural risk factors may be more likely to require enhanced observation, making privacy, consent, dignity and acceptability particularly important when video‐based monitoring is used. Although several included studies examined staff acceptance, reporting on patient consent processes, equity, and patient and family experiences was limited. This gap is consistent with recent evidence showing that research on remote video monitoring has predominantly focused on staff perspectives, while patient, resident and family experiences remain underrepresented [29]. Patients with impaired decision‐making capacity may require appropriate surrogate involvement, while language or communication barriers require accessible and culturally appropriate consent processes. Further research should therefore examine equity, consent, privacy, and patient‐ and family‐reported acceptability, particularly across diverse and non‐U.S. settings.

### 4.6. Implications for Nursing Management

The findings have several implications for nurse managers considering Fall TIPS or video‐based monitoring. Because the two approaches operate through different mechanisms, intervention selection should be based on local patient needs, staffing models, workflow, technological infrastructure and implementation capacity rather than assuming that the approaches are interchangeable. For Fall TIPS, nurse managers have an important role in supporting staff education, patient and family engagement, consistent use of tailored prevention plans and ongoing adherence monitoring. For video‐based monitoring, management priorities include clear patient‐selection criteria, defined escalation and communication pathways, staff competency, privacy and consent procedures, and reliable bedside response processes.

Economic evidence should be interpreted cautiously because most studies report partial and setting‐specific cost or resource‐use outcomes. Nurse managers should therefore evaluate local implementation costs, changes in sitter utilisation, fall and injury outcomes, staff workload and acceptability before wider scale‐up. Ongoing audit and feedback can help determine whether the selected approach remains safe, acceptable and sustainable within the local organisation.

## 5. Strengths and Limitations

This systematic review has several strengths. First, it synthesised evidence from 25 studies across multiple care contexts (e.g., acute care, rehabilitation, emergency department and memory‐care), providing a broad view of how Fall TIPS and video‐based monitoring have been applied in practice. Second, the review was conducted and reported in accordance with PRISMA guidelines. Third, methodological quality and risk of bias were assessed using design‐appropriate JBI critical appraisal tools, enabling interpretation of findings in light of study limitations. Fourth, CHEERS 2022 was used to systematically assess the reporting completeness of studies contributing economic evidence, while the CHEC‐List was additionally applied to the single full economic evaluation. The findings were interpreted in light of the study‐level methodological appraisal, study designs, heterogeneity and consistency of the available evidence.

The review also has several limitations. The evidence base was dominated by U.S.‐based, quasi‐experimental and quality‐improvement studies, with substantial heterogeneity in outcome measures and follow‐up periods, limiting generalisability and precluding pooled quantitative synthesis. Economic reporting was often incomplete, limiting cross‐study comparability and the transferability of economic findings across healthcare settings.

Although the search strategy was developed with a health‐sciences information specialist and covered major biomedical, nursing, and multidisciplinary databases, Embase, the Cochrane Library, IEEE Xplore, ProQuest Dissertations and Theses, and dedicated grey‐literature sources were not searched. In addition, the review was restricted to English‐language studies, which may have introduced language and publication bias.

## 6. Conclusions

Evidence for Fall TIPS generally indicated reductions in falls and injurious falls in acute‐care settings. Video‐based monitoring also showed potential benefits across several delivery models, including reductions in falls and decreased reliance on one‐to‐one sitters, although the findings were more variable and were based predominantly on non‐randomised studies. The findings for the two intervention classes should therefore be interpreted separately.

Economic outcomes were largely limited to cost avoidance, sitter‐hour substitution, budget impact and return‐on‐investment estimates. Only one study conducted a full economic evaluation. Because costing methods, outcome denominators, price years and resource components were reported inconsistently, the economic findings should be interpreted as setting‐specific and do not support firm conclusions regarding cost‐effectiveness.

Successful implementation depends on appropriate patient selection, staff engagement, standardised workflows, and reliable communication and escalation protocols. Future research should use stronger controlled designs, longer follow‐up periods, standardised outcome measures and transparent economic methods. Greater attention is also needed to consent, privacy, equity, and patient and family acceptability, particularly in non‐U.S. and resource‐constrained settings.

## Author Contributions

Fatma Al Jabri, Hannele Turunen and Tarja Tervo‐Heikkinen conceptualised and designed the study. Fatma Al Jabri, Hannele Turunen and Javkhlanbayar Dorjdagva were involved in data acquisition, data analysis and interpretation. Fatma Al Jabri drafted the manuscript. Fatma Al Jabri, Hannele Turunen, Javkhlanbayar Dorjdagva, Minna Mykkänen, Taina Kalliomäki, Antti Hedman and Tarja Tervo‐Heikkinen reviewed the paper and revised it for important intellectual content.

## Funding

The study was funded by the Research Center for Nursing Science and Social & Health Management, Wellbeing Services County of North Savo, Internal Development Funding (Grant No: 1H0029).

## Conflicts of Interest

The authors declare no conflicts of interest.

## Supporting Information

Additional supporting information can be found online in the Supporting Information section.

## Supporting information


**Supporting Information** The supporting information include PRISMA 2020 checklist (Supporting file 1), search string (Supporting file 2), methodological quality appraisal (Supporting file 3), harmonised outcomes (stratified by outcome unit) (Supporting file 4), consolidated health economic evaluation reporting standards checklist (Supporting file 5), Evers CHEC‐List of quality of full economic evaluations (Supporting file 6), and companion and overlapping publications (Supporting file 7), providing detailed data and analysis supporting the main text. These materials are available in the Supporting Section.

## Data Availability

All data obtained from the analysis of the articles included in this review will be provided upon request by the corresponding author.
