# Supplementary material for: Fall Prevention Using the Fall TIPS Toolkit and Video‐Based Monitoring: A Systematic Review of Clinical Effectiveness and Economic Outcomes
Source: J Nurs Manag. 2026 Nov 10;2026:9948784. doi: 10.1155/jonm/9948784 (PMC13752480; doi:10.1155/jonm/9948784)
Supplement: Supplementary file 1 [file JONM-2026-9948784-s001.zip › Supplementary File 7.docx]

**Supplementary File 7: Companion and Overlapping Publications**

| **Publication Cluster** | **Health system** | **Setting/ Units** | **Calender period** | **Intervention** | **Primary Effectiveness Publication** | **Role of Companion Publication** |
| --- | --- | --- | --- | --- | --- | --- |
| Sand-Jecklin et al. (2016, 2019) | University Medical Center (US) | Medical-surgical units | 2013-2014 | Centralized video-based monitoring | Sand-Jecklin et al. (2016) | Sand-Jecklin et al. (2019): process/standardisation evidence only; not counted as independent clinical-effectiveness evidence. |
| Dykes et al. (2017, 2020, 2021, 2023) | Fall TIPS program | Acute care units | 2015-2023 | Fall TIPS toolkit | **Dykes et al. (2020)** | Dykes et al. (2017): pilot/implementation and adherence; Dykes et al. (2021): acceptability/time; Dykes et al. (2023): economic evidence. Companion publications were not counted as independent clinical-effectiveness evidence. |
