# Supplementary material for: Fall Prevention Using the Fall TIPS Toolkit and Video‐Based Monitoring: A Systematic Review of Clinical Effectiveness and Economic Outcomes
Source: J Nurs Manag. 2026 Nov 10;2026:9948784. doi: 10.1155/jonm/9948784 (PMC13752480; doi:10.1155/jonm/9948784)
Supplement: Supplementary file 1 [file JONM-2026-9948784-s001.zip › Supplementary File 2.docx]

**Supplementary File 2: Search String**

| **Databases** | **Term** | **Limiters** | **Records retrieved** |
| --- | --- | --- | --- |
| Web of Science | **("camera monitoring" OR "fall prevention" OR "fall TIPS model" OR "pattern recognition system" OR "video monitoring") AND ("healthcare settings" OR "hospital") AND cost*** | Language: English  Document type: Article  Publication years: 2014-2026 | 159 |
| Scopus | TITLE-ABS-KEY ( ( "camera monitoring" OR "fall prevention" OR "fall TIPS model" OR "pattern recognition system" OR "video monitoring" ) AND ( "healthcare settings" OR "hospital" ) AND cost* ) | Language: English  Document type: Article  Subject area: Health Professionals or nursing  Publication years: 2014-2026 | 87 |
| CINAHL | ("camera monitoring" OR "fall prevention" OR "fall TIPS model" OR "pattern recognition system" OR "video monitoring") AND ("healthcare settings" OR "hospital") AND cost* | Language: English  Publication years: 2014-2026  Publication type: Academic journal | 98 |
| PubMed | **("camera monitoring" OR "fall prevention" OR "fall TIPS model" OR "pattern recognition system" OR "video monitoring") AND ("healthcare settings" OR "hospital") AND cost*** | Language: English  Publication years: 2014-2026  Text availability: Abstract, full-text  Species: Humans | 197 |
