# Supplementary material for: Fall Prevention Using the Fall TIPS Toolkit and Video‐Based Monitoring: A Systematic Review of Clinical Effectiveness and Economic Outcomes
Source: J Nurs Manag. 2026 Nov 10;2026:9948784. doi: 10.1155/jonm/9948784 (PMC13752480; doi:10.1155/jonm/9948784)
Supplement: Supplementary file 1 [file JONM-2026-9948784-s001.zip › Supplementary File 3.docx]

**Supplementary File 3: Quality Appraisal**

JBI critical appraisal checklist items were rated as Yes, No, Unclear, or Not Applicable. While Mixed-Methods Appraisal Tool (MMAT) items were rated as Yes, No, Can't tell

**JBI - Cross-sectional designs studies (n = 4)**

| **Criteria** | **Dykes et al., 2021** | **Hebb et al., 2021** | **Quigley et al. (2019)** | **Ergai et al. (2023)** |
| --- | --- | --- | --- | --- |
| 1. Were the criteria for inclusion in the sample clearly defined? | Y | Y | Y | Y |
| 2. Were the study subjects and the setting described in detail? | Y | Y | Y | Y |
| 3. Was the exposure measured in a valid and reliable way? | Y | Y | Y | Y |
| 4. Were objective, standard criteria used for measurement of the condition? | Y | NA | UC | NA |
| 5. Were confounding factors identified? | Y | N | Y | UC |
| 6. Were strategies to deal with confounding factors stated? | Y | N | N | N |
| 7. Were the outcomes measured in a valid and reliable way? | Y | Y | UC | Y |
| 8. Was appropriate statistical analysis used? | Y | Y | Y | Y |

**JBI - Quasi-experimental designs studies (n = 14)**

| **Criteria** | **Cook et al. (2020)** | **Dykes et al. (2020)** | **Jones et al. (2021** | **Davis & Carter-Templeton (2020)** | **Quigley et al. (2021)** | **Cournan et al. (2018)** | **Purvis et al. (2018)** | **Dykes et al. (2017)** | **Woltsche et al. (2022)** | **Sand-Jecklin et al. (2016)** | **Bayen et al. (2017)** | **Burtson & Vento (2015)** | **Votruba et al. (2016)** | **Kowalski et al. (2018)** |
| --- | --- | --- | --- | --- | --- | --- | --- | --- | --- | --- | --- | --- | --- | --- |
| 1.Is it clear in the study what is the “cause” and what is the “effect” (i.e. there is no confusion about which variable comes first)? | Y | Y | Y | Y | Y | Y | Y | Y | Y | Y | Y | Y | Y | Y |
| 2. Was there a control group? | N | Y | Y | N | N | N | N | N | N | N | N | Y | Y | N |
| 3. Were participants included in any comparisons similar? | UC | Y | Y | UC | UC | UC | UC | Y | N | Y | NA | Y | Y | Y |
| 4. Were the participants included in any comparisons receiving similar treatment/care, other than the exposure or intervention of interest? | UC | Y | Y | UC | Y | UC | UC | Y | UC | Y | Y | N | N | Y |
| 5 Were there multiple measurements of the outcome, both pre and post the intervention/exposure? | Y | Y | N | N | Y | Y | Y | Y | Y | Y | Y | Y | Y | N |
| 6 Were the outcomes of participants included in any comparisons measured in the same way? | Y | Y | Y | Y | Y | Y | Y | Y | Y | Y | Y | Y | Y | Y |
| 7 Were outcomes measured in a reliable way? | Y | Y | Y | Y | Y | Y | Y | Y | Y | Y | Y | Y | Y | Y |
| 8 Was follow-up complete and if not, were differences between groups in terms of their follow-up adequately described and analyzed? | Y | Y | Y | NA | NA | NA | NA | NA | NA | NA | NA | NA | NA | NA |
| 9 Was appropriate statistical analysis used? | Y | Y | Y | Y | Y | Y | Y | Y | Y | Y | N | N | Y | Y |

**JBI - Qualitative designs studies (n = 2)**

| **Criteria** | **Klymko et al. (2016)** | **Medeiros & Gonçalves (2024)** |
| --- | --- | --- |
| 1. Is there congruity between the stated philosophical perspective and the research methodology? | UC | UC |
| 2. Is there congruity between the research methodology and the research question or objectives? | Y | Y |
| 3. Is there congruity between the research methodology and the methods used to collect data? | Y | Y |
| 4. Is there congruity between the research methodology and the representation and analysis of data? | Y | Y |
| 5. Is there congruity between the research methodology and the interpretation of results? | Y | Y |
| 6. Is there a statement locating the researcher culturally or theoretically? | Y | UC |
| 7. Is the influence of the researcher on the research, and vice- versa, addressed? | Y | Y |
| 8. Are participants, and their voices, adequately represented? | Y | Y |
| 9. Is the research ethical according to current criteria or, for recent studies, and is there evidence of ethical approval by an appropriate body? | Y | Y |
| 10. Do the conclusions drawn in the research report flow from the analysis, or interpretation, of the data? | Y | Y |

**JBI - Randomised Controlled Trial (n = 1)**

| **Criteria** | **Daley et al. (2020)** |
| --- | --- |
| 1. Was true randomization used for assignment of participants to treatment groups? | Y |
| 2. Was allocation to treatment groups concealed? | N |
| 3. Were treatment groups similar at the baseline? | UC |
| 4. Were participants blind to treatment assignment? | N |
| 5. Were those delivering treatment blind to treatment assignment? | N |
| 6. Were outcome assessors blind to treatment assignment? | N |
| 7. Were treatment groups treated identically other than the intervention of interest? | Y |
| 8. Was follow-up complete, and if not, were differences between groups in terms of their follow-up adequately described and analyzed? | Y |
| 9. Were participants analyzed in the groups to which they were randomized (intention-to-treat analysis)? | UC |
| 10. Were outcomes measured in the same way for treatment groups? | Y |
| 11. Were outcomes measured in a reliable way? | Y |
| 12. Was appropriate statistical analysis used? | Y |
| 13. Was the trial design appropriate, and were any deviations from the standard RCT design (e.g., cluster randomization, crossover design) accounted for in the conduct and analysis of the trial? | UC |

**JBI - Economic Evaluation (n = 1)**

| **Criteria** | **Dykes et al. (2023)** |
| --- | --- |
| 1. Is there a well-defined question? | Y |
| 2. Is there comprehensive description of alternatives? | Y |
| 3. Are all important and relevant costs and outcomes for each alternative identified? | Y |
| 4. Has clinical effectiveness been established? | Y |
| 5. Are costs and outcomes measured accurately? | Y |
| 6. Are costs and outcomes valued credibly? | Y |
| 7. Are costs and outcomes adjusted for differential timing? | UC |
| 8. Is there an incremental analysis of costs and consequences? | Y |
| 9. Were sensitivity analyses conducted to investigate uncertainty in estimates of cost or consequences? | Y |
| 10. Do study results include all issues of concern to users? | Y |
| 11. Are the results generalizable to the setting of interest in the review? | Y |

**JBI – Cohort Studies (n = 2)**

| **Criteria** | **Sosa et al. (2024)** | **Kalafus et al. (2025)** |
| --- | --- | --- |
| 1. Were the two groups similar and recruited from the same population? | Y | Y |
| 2. Were the exposures measured similarly to assign people to both exposed and unexposed groups? | Y | Y |
| 3. Was the exposure measured in a valid and reliable way? | Y | Y |
| 4. Were confounding factors identified? | Y | Y |
| 5. Were strategies to deal with confounding factors stated? | Y | N |
| 6. Were the groups/participants free of the outcome at the start of the study (or at the moment of exposure)? | Y | Y |
| 7. Were the outcomes measured in a valid and reliable way? | Y | Y |
| 8. Was the follow up time reported and sufficient to be long enough for outcomes to occur? | Y | Y |
| 9. Was follow up complete, and if not, were the reasons to loss to follow up described and explored? | UC | UC |
| 10. Were strategies to address incomplete follow up utilized? | NA | NA |
| 11. Was appropriate statistical analysis used? | Y | Y |

**Mixed-methods studies (n = 1)**

| **Criteria** | **Sand-Jecklin et al. (2019** |
| --- | --- |
| S1. Are there clear research questions? | Y |
| S2. Do the collected data allow to address the research questions? | Y |
| 1. Is there an adequate rationale for using a mixed methods design to address the research question? | Can't tell |
| 2. Are the different components of the study effectively integrated to answer the research question? | Y |
| 3. Are the outputs of the integration of qualitative and quantitative components adequately interpreted? | Y |
| 4. Are divergences and inconsistencies between quantitative and qualitative results adequately addressed? | Y |
| 5. Do the different components of the study adhere to the quality criteria of each tradition of the methods involved? | Can't tell |
