# Supplementary material for: Fall Prevention Using the Fall TIPS Toolkit and Video‐Based Monitoring: A Systematic Review of Clinical Effectiveness and Economic Outcomes
Source: J Nurs Manag. 2026 Nov 10;2026:9948784. doi: 10.1155/jonm/9948784 (PMC13752480; doi:10.1155/jonm/9948784)
Supplement: Supplementary file 1 [file JONM-2026-9948784-s001.zip › Supplementary File 4.docx]

# Supplementary File 4: Harmonized Outcomes (Stratified by Outcome Unit)

| **Study** | **Setting** | **Design** | **Outcome (unit)** | **Baseline** | **Follow-up** | **Absolute change** | **Relative change** | **Effect direction** |
| --- | --- | --- | --- | --- | --- | --- | --- | --- |
| **Falls / Injurious falls per 1,000 patient-days** | | | | | | | | |
| Fall TIPS | | | | | | | | |
| Dykes et al. (2017) | Acute care units (USA) | QI pilot / pre–post | Injurious falls (/1,000 patient-days) | 1.00 | 0.54 | -0.46 | -46.0% | ↓ |
| Dykes et al. (2020) | Acute care medical units (USA) | Stepped-wedge ITS | Falls (/1,000 patient-days) | NR | NR | NR | -15% | ↓ |
|  |  |  | Injurious falls (/1,000 patient-days) | NR | NR | NR | -34% | ↓ |
| Video monitoring | | | | | | | | |
| Cournan et al. (2018) | Rehabilitation hospital (USA) | Sequential cohort (pre–post) | Falls (/1,000 patient-days) | 6.34 | 5.09 | -1.25 | -19.7% | ↓ |
| Daley et al. (2020) | Neuro-medical unit (USA) | Randomized trial | Falls (/1,000 patient-days) — ESS + rounder | NR | 1.02 | NR | NR | ↔ |
|  |  |  | Falls (/1,000 patient-days) — ESS only | NR | 3.05 | NR | NR | ↔ |
| Kalafus et al. (2025) | Long-term acute care hospital (USA) | Prospective observational cohort | Falls (/1,000 patient-days) | 4.9 | 3.8 | -1.1 | -22.4% | ↓ |
| Davis & Carter-Templeton (2020) | Orthopedic unit (USA) | QI before–after | Falls (/1,000 patient-days) | 2.6 | 0.0 | -2.6 | -100% | ↓ |
| Sand-Jecklin et al. (2016) | Med-surg units (USA) | Quasi-experimental (pre–post) | Falls (/1,000 patient-days) | 3.9 | 2.8 | -1.1 | -28.2% | ↓ |
| **Falls / Injurious falls per 1,000 visits (ED)** | | | | | | | | |
| Multifactorial bundle incl. VM | | | | | | | | |
| Cook et al. (2020) | Emergency Department (USA) | QI (pre–post over time) | Falls (/1,000 visits) | 0.73 | 0.55 | -0.18 | -24.7% | ↓ |
|  |  |  | Falls with injury (/1,000 visits) | 0.09 | 0.03 | -0.06 | -66.7% | ↓ |
| **Falls / Injurious falls per 1,000 admissions** | | | | | | | | |
| Automated/electronic surveillance (AVMS) | | | | | | | | |
| Jones et al. (2021) | Rural hospitals (USA) | One-group pre–post | Falls (/1,000 admissions) | NR | NR | NR | NR | ↓ |
| **Falls per discharge** | | | | | | | | |
| Video monitoring (telesitter/VPR) | | | | | | | | |
| Votruba et al. (2016) | Acute units (USA) | Prospective QI (pre–post) | Falls per discharge (%) | 1.7% | 1.1% | -0.6 pp | -35.3% | ↓ |
| **Percent-based fall outcomes** | | | | | | | | |
| Video monitoring (CVM + incident review) | | | | | | | | |
| Bayen et al. (2017) | Memory-care dementia facility (USA) | Pilot before–after | Falls (% reduction) | NR | NR | NR | -79% | ↓ |
| Video monitoring (hybrid two-way VM + sitters) | | | | | | | | |
| Sosa et al. (2024) | Acute hospital (USA) | Retrospective pre–post | % patients with falls | 3.5% | 2.7% | -0.8 pp | -22.9% | ↓ |
| **Relative effects without baseline/follow-up values** | | | | | | | | |
| Fall TIPS | | | | | | | | |
| Dykes et al. (2021) | Acute care hospitals (USA) | Survey/ implementation | Falls (relative change) | NR | NR | NR | -25% | ↓ |
|  |  |  | Injurious falls (relative change) | NR | NR | NR | -34% | ↓ |
| Dykes et al. (2023) | Acute care (USA) | Implementation + cost–benefit | Falls (relative change) | NR | NR | NR | -19% | ↓ |
|  |  |  | Injurious falls (relative change) | NR | NR | NR | -20% | ↓ |
| Video monitoring | | | | | | | | |
| Ergai et al. (2023) | Acute/ICU (USA) | Quasi-experimental pre–post | Falls with injury (relative change) | NR | NR | NR | -39.15% | ↓ |
| Quigley et al. (2022) | Adult inpatients (USA) | QI pre–post | Fall rate (relative change) | NR | NR | NR | -14% | ↓ |
|  |  |  | Injury rate (relative change) | NR | NR | NR | -6% | ↓ |
| Kowalski et al. (2018) | Inpatient facility (USA) | Budget-impact/projection | Fall-related costs (relative change) | NR | NR | NR | -50% (cost) | ↓ |
| Burtson & Vento (2015) | Acute hospital system (USA) | Program evaluation | Falls vs national benchmark | NR | NR | NR | Comparable | ↔ |
| **Studies without quantified fall outcome effects** | | | | | | | | |
| Fall TIPS | | | | | | | | |
| Medeiros & Gonçalves (2024) | Teaching hospital (Brazil) | Evaluability study | Proposed indicators (no effect estimate) | NA | NA | NA | NA | NA |
| Video monitoring | | | | | | | | |
| Klymko et al. (2016) | Hospital context (USA) | Qualitative focus groups | No quantified fall outcomes | NA | NA | NA | NA | NA |
| Hebb et al. (2021) | Acute care units (USA) | Survey | Acceptability/perceived effectiveness | NA | NA | NA | NA | ↔ |
| Purvis et al. (2018) | Academic medical center (USA) | QI implementation | Falls (counts; VM vs PSA period) | 8 falls (PSA period) | 1 fall (VM period) | -7 | -87.5% | ↓ |
| Quigley et al. (2019) | Multi-hospital program (USA) | Program evaluation | Falls/adverse events (aggregate) | NR | NR | NR | NR | ↓ |
| Sand-Jecklin et al. (2019) | Acute hospital (USA) | Process evaluation | Process/standardization (no new effect estimate) | NA | NA | NA | NA | NA |
| Woltsche et al. (2022) | Aged care wards (Australia) | Prospective cohort/QI | Falls (/1,000 bed-days) — overnight | NR | NR | NR | NR | ↓ |

**Abbreviations:** AVMS = automated video monitoring system; CVM = continuous video monitoring; ED = emergency department; ESS = electronic surveillance system; ICU = intensive care unit; ITS = interrupted time series; NA = not applicable; NR = not reported; pp = percentage points; PSA = patient safety attendant; QI = quality improvement; VM = video monitoring; VPR = virtual patient monitoring.

**Effect direction:** ↓ decrease; ↔ no meaningful change.
