# Supplementary material for: Fall Prevention Using the Fall TIPS Toolkit and Video‐Based Monitoring: A Systematic Review of Clinical Effectiveness and Economic Outcomes
Source: J Nurs Manag. 2026 Nov 10;2026:9948784. doi: 10.1155/jonm/9948784 (PMC13752480; doi:10.1155/jonm/9948784)
Supplement: Supplementary file 1 [file JONM-2026-9948784-s001.zip › Supplementary File 5.docx]

**Supplementary File 5: Consolidated health economic evaluation reporting standards checklist**

***Note: R = Reported; NR = Not reported.***

| **Section Item** | **Bayen et al. (2017)** | **Burtson & Vento (2015)** | **Cook et al., (2020)** | **Cournan et al. (2018)** | **Daley et al. (2020)** | **Davis & Carter-Templeton (2020)** | **Dykes et al. (2021)** | **Dykes et al. (2023)** | **Dykes et al. (2020)** | **Dykes et al. (2017)** | **Ergai et al. (2023)** | **Hebb et al. (2021)** |
| --- | --- | --- | --- | --- | --- | --- | --- | --- | --- | --- | --- | --- |
| Title | NR | NR | NR | NR | NR | R | NR | R | NR | NR | NR | NR |
| Abstract | R | R | R | R | R | R | R | R | R | R | R | R |
| Background and objectives | R | R | R | R | R | R | R | R | R | R | R | R |
| Health economic analysis plan | NR | R | NR | R | NR | NR | NR | R | NR | NR | NR | NR |
| Study population | R | R | R | R | R | R | R | R | R | R | R | R |
| Setting and location | R | R | R | R | R | R | R | R | R | R | R | R |
| Comparators | R | R | R | R | R | R | R | R | R | R | R | R |
| Perspective | R | R | NR | R | R | R | R | R | R | R | R | R |
| Time horizon | R | R | R | R | R | R | R | R | R | R | R | R |
| Discount rate | NR | R | NR | NR | NR | NR | NR | R | NR | NR | NR | NR |
| Selection of outcomes | R | R | R | R | R | R | R | R | R | R | R | R |
| Measurement of outcomes | R | R | R | R | R | R | R | R | R | R | R | R |
| Valuation of outcomes | R | R | NR | R | R | R | R | R | R | R | R | R |
| Measurement and valuation of resources and costs | NR | R | NR | R | NR | R | NR | R | NR | R | NR | NR |
| Currency, price date, and conversion | NR | R | NR | NR | NR | NR | NR | R | NR | R | NR | NR |
| Rationale and description of model | NR | R | NR | NR | R | NR | R | R | NR | R | NR | R |
| Analytics and assumptions | R | R | R | R | R | R | R | R | R | R | R | R |
| Characterizing heterogeneity | R | R | NR | R | R | NR | R | R | R | R | NR | R |
| Characterizing distributional effects | R | R | NR | NR | R | R | R | R | R | R | NR | R |
| Characterizing uncertainty | R | R | R | R | R | R | R | R | R | R | R | R |
| Approach to engagement with patients and others affected by the study | R | R | R | R | R | R | R | R | R | R | R | R |
| Study parameters | R | R | R | R | R | R | R | R | R | R | R | R |
| Summary of main results | R | R | R | R | R | R | R | R | R | R | R | R |
| Effect of uncertainty | R | R | R | R | R | R | NR | R | R | R | R | R |
| Effect of engagement with patients and others affected by the study | R | R | NR | R | R | R | R | R | NR | R | R | R |
| Study findings, limitations, generalizability, and current knowledge | R | R | R | R | R | R | R | R | R | R | R | R |
| Source of fundings | R | NR | NR | R | NR | NR | R | R | R | R | NR | NR |
| Conflict of interest | R | R | R | R | R | R | R | R | R | R | R | R |
| **Total** | **22** | **26** | **16** | **23** | **22** | **22** | **22** | **28** | **21** | **25** | **19** | **22** |

| **Section Item** | **Jones et al. (2021)** | Kalafus et al., 2025 | **Klymko et al. (2016)** | **Kowalski et al. (2018)** | **Medeiros & Gonçalves (2024)** | **Purvis et al. (2018)** | **Quigley et al. (2022)** | **Quigley et al. (2019)** | **Sand-Jecklin et al. (2016)** | **Sand-Jecklin et al. (2018)** | **Sosa et al. (2024)** | **Votruba et al. (2016)** | **Woltsche et al. (2022)** |
| --- | --- | --- | --- | --- | --- | --- | --- | --- | --- | --- | --- | --- | --- |
| Title | NR | NR | NR | R | NR | NR | NR | NR | NR | NR | R | R | NR |
| Abstract | R | R | R | R | R | R | R | R | R | R | R | R | R |
| Background and objectives | R | R | R | R | R | R | R | R | R | R | R | R | R |
| Health economic analysis plan | NR | R | NR | R | NR | R | R | NR | NR | NR | R | R | NR |
| Study population | R | R | R | R | R | R | R | R | R | R | R | R | R |
| Setting and location | R | R | R | R | R | R | R | R | R | R | R | R | R |
| Comparators | R | R | NR | R | NR | R | R | R | R | NR | R | R | R |
| Perspective | R | R | R | R | R | R | R | R | R | R | R | R | R |
| Time horizon | R | R | R | R | R | R | R | R | R | R | R | R | R |
| Discount rate | NR | NR | NR | R | NR | R | R | NR | NR | NR | R | R | NR |
| Selection of outcomes | R | R | R | R | NR | R | R | R | R | R | R | R | R |
| Measurement of outcomes | R | R | R | R | NR | R | R | R | R | R | R | R | R |
| Valuation of outcomes | R | R | NR | R | R | R | R | R | R | R | R | R | R |
| Measurement and valuation of resources and costs | NR | R | NR | R | R | R | R | NR | R | NR | R | R | R |
| Currency, price date, and conversion | NR | R | NR | R | NR | R | NR | R | R | NR | R | R | R |
| Rationale and description of model | R | NR | NR | R | R | R | R | NR | R | NR | R | NR | NR |
| Analytics and assumptions | R | R | NR | R | R | NR | R | NR | R | NR | R | R | R |
| Characterizing heterogeneity | R | NR | NR | R | NR | R | R | R | R | R | R | NR | R |
| Characterizing distributional effects | R | NR | R | R | NR | R | NR | R | R | R | R | R | R |
| Characterizing uncertainty | R | NR | R | R | NR | R | R | R | R | R | R | R | R |
| Approach to engagement with patients and others affected by the study | R | R | R | R | R | R | R | R | R | R | R | R | R |
| Study parameters | R | R | R | R | NR | R | R | R | R | R | R |  | R |
| Summary of main results | R | R | R | R | R | R | R | R | R | R | R | R | R |
| Effect of uncertainty | R | NR | R | R | NR | R | R | R | R | R | R | R | R |
| Effect of engagement with patients and others affected by the study | R | NR | R | R | R | R | NR | R | R | R | R | R | R |
| Study findings, limitations, generalizability, and current knowledge | R | R | R | R | R | R | R | R | R | R | R | R | R |
| Source of fundings | R | R | NR | R | NR | NR | R | NR | NR | NR | R | NR | R |
| Conflict of interest | R | R | NR | NR | NR | R | R | R | R | R | NR | NR | R |
| **Total** | **23** | **20** | **16** | **27** | **14** | **25** | **24** | **21** | **24** | **19** | **27** | **24** | **24** |
