# Supplementary material for: Fall Prevention Using the Fall TIPS Toolkit and Video‐Based Monitoring: A Systematic Review of Clinical Effectiveness and Economic Outcomes
Source: J Nurs Manag. 2026 Nov 10;2026:9948784. doi: 10.1155/jonm/9948784 (PMC13752480; doi:10.1155/jonm/9948784)
Supplement: Supplementary file 1 [file JONM-2026-9948784-s001.zip › Supplementary File 6.docx]

**Supplementary File 6: Evers CHEC-List of quality of full economic evaluations**

| **Items** | **Dykes et al., 2023** |
| --- | --- |
| 1. Is the study population clearly described? | Y |
| 2. Are competing alternatives clearly described? | Y |
| 3. Is a well-defined research question posed in answerable form? | Y |
| 4. Is the economic study design appropriate to the stated objective? | Y |
| 5. Is the chosen time horizon appropriate to include relevant costs and consequences? | Y |
| 6. Is the actual perspective chosen appropriate? | Y |
| 7. Are all relevant costs for each alternative identified? | Y |
| 8. Are all costs measured appropriately in physical units? | Y |
| 9. Are all costs valued appropriately? | Y |
| 10. Are all important and relevant outcomes of alternatives performed? | Y |
| 11. Are all outcomes measured appropriately? | Y |
| 12. Are outcomes valued appropriately? | Y |
| 13. Is an incremental analysis of costs and outcomes of alternative performed? | Y |
| 14. Are all future costs and outcomes discounted appropriately? | NA |
| 15. Are all important variables, whose values are uncertain, appropriately subjected to sensitivity analysis? | Y |
| 16. Do the conclusions follow from the data reported? | Y |
| 17. Does the study discuss the generalisability of the results to other settings and patient and client groups? | Y |
| 18. Does the article indicate that there is no potential conflict of interest of study researcher and funder? | Y |
| 19. Are ethical and distributional issues discussed appropriately? | Y |
| Summary score | 18/19 |
